# Laboratory study of tissue repair of resin-based endodontic sealers in critical surgical defects

**DOI:** 10.1590/1678-7757-2022-0108

**Published:** 2022-08-01

**Authors:** Guilherme Ferreira da SILVA, Letycia Accioly Simões COELHO, Vanessa Abreu Sanches COSTA, Letícia Citelli CONTI, Ana Carolina de Almeida LIMA, Gabriela Cristina de Santi SODRÉ, Mateus Rinaldi Lucio MARTINS, Marco Antonio Hungaro DUARTE, Rodrigo Ricci VIVAN

**Affiliations:** 1 Unisagrado Bauru SP Brasil Centro Universitário Sagrado Coração, Unisagrado , Curso de Odontologia, Bauru , SP , Brasil .; 2 Universidade de São Paulo Faculdade de Odontologia de Bauru Departamento de Dentística, Endodontia e Materiais Odontológicos Bauru SP Brasil Universidade de São Paulo , Faculdade de Odontologia de Bauru , Departamento de Dentística, Endodontia e Materiais Odontológicos , Bauru , SP , Brasil .

**Keywords:** Inflammation, Materials testing, Root canal filling materials

## Abstract

Filling materials should be restricted to the root canal space. However, sometimes it is impossible to control the apical extrusion, in this case, the fate of the filling material and the result of the treatment will depend on its physicochemical properties and biocompatibility.

**Objective:**

To evaluate the tissue response and bone repair capacity of endodontic sealers that were implanted in the calvaria of Wistar rats, forming the groups (n=16): AH Plus and Sealer Plus, compared to the clot group.

**Methodology:**

On days 30 and 60, the animals were euthanized, the calvaria was removed and processed for hematoxylin-eosin, immunohistochemistry for collagen type I, *Picrosirus* red and microtomographic analysis. Data were subjected to ANOVA and Tuckey tests (p<0.05).

**Results:**

At 30 days, all groups showed an intense inflammatory reaction (p>0.05). At 60 days, the AH Plus and Sealer Plus maintained an intense inflammatory infiltrate compared to the clot group (p<0.05). We observed immunopositive areas for type I collagen in all groups at 30 days and 60 days (p>0.05). We observed more red collagen fibers for the Sealer Plus compared to the clot group at 30 days (p<0.05). Considering the total fibers, the clot group at 30 days compared to 60 days after surgery showed an increase in the amount of matrix (p<0.05). There were no statistical differences between groups for green and yellow fibers (p>0.05). Regarding morphometric parameters, at 30 days, the newly formed bone volume and number of bone trabeculae were higher in the groups with sealers compared to the clot group (p<0.05). At 60 days, AH Plus and Sealer Plus showed greater bone neoformation compared to the clot group (p<0.05).

**Conclusions:**

Despite AH Plus and Sealer Plus induced an intense inflammatory reaction, they can be considered biocompatible materials, since they allowed bone repair.

## Introduction

Endodontic treatment depends on infection control by cleaning, shaping and filling the root canal system. ^1 , 2^ The root canal filling materials must show biocompatibility and sealing capacity to prevent both colonization and reinfection by pathogenic microorganisms, preventing communication and irritation to the periapex. ^2 , 3 , 4^

Filling materials must be restricted to the space of the root canal. ^5 , 6^ However, sometimes it is impossible to control the application of the material and an apical extrusion occurs. ^7^ In these cases, the fate of the filling material will depend on its solubility in tissue fluids, the susceptibility to phagocytosis, and its biocompatibility. ^7^ The overfilled material may act as a foreign body, inducing histological reactions ranging from simple periradicular inflammation to necrosis of the periodontal ligament. ^8^

AH Plus (Dentsply Maillefer, Ballaigues, Vaud, Switzerland) is an epoxy resin-based sealer and considered the gold standard endodontic sealer due to its physicochemical properties (setting time, flow, film thickness, dimensional stability, radiopacity and solubility), ^9 , 10 , 11^ biocompatibility, ^12 , 13^ sealing ability ^14 , 15^ and satisfactory antimicrobial activity. ^16^ AH Plus sealer is commonly used in clinical practice and experimental studies in animals that examined periapical repair against this material reported good biological activity. ^17 , 18 , 19 , 20^

Sealer Plus (MK LIFE – Michel E. Klymus Me, Porto Alegre, Rio Grande do Sul, Brazil) consists of a sealer based on epoxy resin too. Its composition is similar to AH Plus, containing calcium tungstate, zirconium oxide and radiopacifiers. The main difference is the presence of calcium hydroxide in the base and catalyst pastes of Sealer Plus. ^21^ According to the manufacturer, the sealer has exceptional viscosity, penetrability and low shrinkage. This endodontic sealer proved to be biocompatible when analyzed histologically in subcutaneous tissue of rats. ^21^ Other studies have evaluated its physical-chemical properties, ^22 - 25^ proving the bioactive potential of this material. Its antimicrobial activity was tested in the study by Silva, et al. ^26^ (2020). However, the literature lacks data on the biological behavior of this material in contact with organic tissues, as in cases of periapical extravasation.

Therefore, we aimed to evaluate the biological response of Sealer Plus in critical surgical defects of rat calvaria by microtomographic (µTC), histological and immunohistochemical analysis. The null hypothesis is that there will be no tissue repair capacity in the presence of resinous endodontic sealers.

## Methodology

### *In vivo* experimental design

The experimental protocol was approved by the Animal Experimentation Ethics Committee (CEUA nº 8239200318).

**Figure 1:**
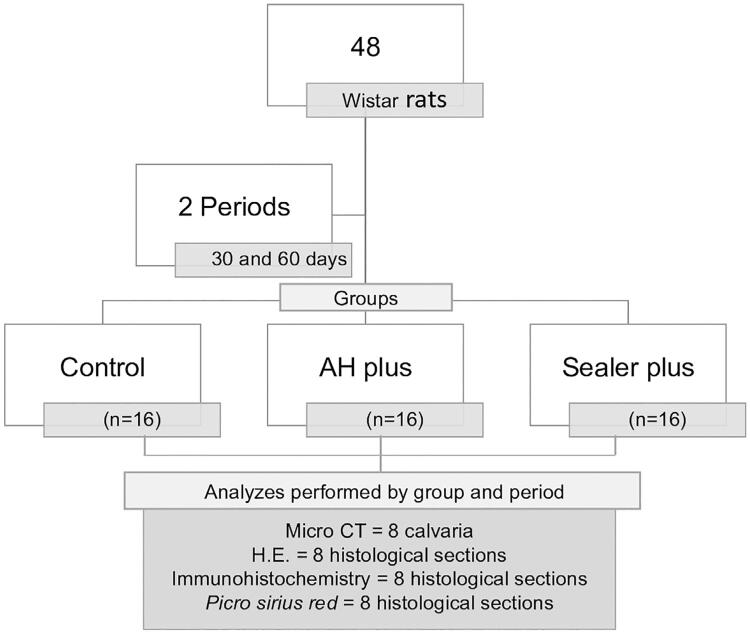
Flowchart showing the experimental stages and their order fulfillment

A total of 48 healthy adult male rats ( *Rattus norvegicus albinus,* Wistar), weighing 450 to 500 g, were randomly divided into three equal groups, comprising the study sample: Clot; Sealer Plus; AH Plus and subdivided into two periods (30 and 60 days) with eight defects per treatment group (n=8), according to Figure 1 .

G * Power v3.1 for Mac (Heinrich Heine, Universität Düsseldorf, Dusseldorf, Germany) and the Wilcoxon-Mann-Whitney test, from the t-test family, were used to estimate the sample. Sample size was estimated based on data from previous study. ^27^ Considering an alpha error of 0.05% and 95% power to recognize a significant difference, a minimum of five animals per group was necessary. Considering possible animal deaths, three more animals were added in each group, resulting in eight rats per group. Random numbers were generated using the standard=rand() function in Microsoft Excel (Microsoft Corporation, Redmond, Washington. USA).

The animals were kept in individual cages, in an environment with a temperature between 22 and 24°C with a controlled light cycle (12 hours light and 12 hours dark) and fed with standard solid food and water *ad libitum* during the experiment.

A senior veterinarian conducted all the nutritional recommendations and was in charge of the care, pre- and postoperative fasting of the animals, in accordance with the guidelines of the National Institutes of Health Guide for the Care and Use of Laboratory Animals, and reported according to the ARRIVE guidelines (Animal Research: Reporting of *in vivo* experiments). ^28 , 29^

### Surgery of the experimental groups

After fasting for a maximum eight hours, the animals were sedated by intramuscular administration of 1% Ketamine Hydrochloride (75 mg/kg) (Vetaset – ZOETIS Indústria de Produtos Veterinários Ltda, Campinas, São Paulo, Brazil) and 2% Xylazine Hydrochloride (10 mg/kg) (Dopaser – Laboratório Calier do Brasil Ltda, São Paulo, São Paulo, Brazil). After anesthesia, trichotomy was performed in the frontoparietal region, the animal was placed in the frontal decubitus, the area was sanitized with topical polyvinylpyrrolidone (PVPI) and a sterile field was applied. Then, surgical access was performed through a “V” shaped incision, the total flap was folded back and removed with the retractors to expose the parietal bone on both sides.

An osteotomy was performed in the median region between the parietals and the internal cortex with a surgical trephine with an internal diameter of 4 mm and an external diameter of 5 mm (Neodent, Curitiba, Paraná, Brazil), with the aid of a low-speed motor. The osteotomized parietal bone was removed and the dura mater was kept intact, leaving a critical size defect of 5 mm in diameter. ^30^

The bone defect was performed in all animals and filled with the respective material of the group in which they belonged: blood clot, AH Plus and Sealer Plus. The sealers were handled according to the manufacturer’s instructions, introduced into insulin syringes (Injex Indústria Cirúrgica Ltda, Ourinhos, São Paulo, Brazil) and 0.2 ml were immediately inserted into the respective cavity. After the graft was completed, the entire flap was repositioned, sutured with a 4-0 nylon thread (Ethicon- Johnson, São José dos Campos, São Paulo, Brazil) using simple interrupted stitches.

The specimens were euthanized in periods of 30 and 60 days, with eight animals from each group per period, by anesthetic overdose with Sodium Thiopental (240 mg/kg), via intraperitoneal (Cristália, Produtos Químicos Farmacêuticos Ltda, Itapira, São Paulo, Brazil).

### Microtomographic analysis

After euthanasia of the 48 specimens, the parietal bone was removed with a margin of three mm of bone tissue around the defect and immediately fixed in 10% buffered formalin (pH 7.2).

All specimens were scanned using a computerized microtomography (SkyScan 1146, Billerica, Massachusetts, New England, USA) at an energy level of 50 Kv and current of 800 µA. Images were captured with a ٢٢.٩ µm pixel camera, with 180° rotation around the vertical axis and 1.0° rotation step. X-rays, filtered with a 0.05 mm aluminum filter and a flat field correction, were taken the day before the scan to correct variations in the camera’s pixel sensitivity.

All images obtained in the scan were reconstructed using NRecon software v.1.6.3, (Bruker microCT). The newly formed bone was analyzed in both experimental periods in microtomographic sections using the CTan and CTvol software to measure the volume, number, separation and thickness of bone trabeculae present in the critical defect with a diameter of five mm, equivalent to the size of the trephine used.

### Histological procedures and analysis

Then, all samples were washed in running water for 24 hours and immersed in 10% ethylenediaminetetraacetic acid (EDTA) for inclusion processing in paraffin. The blocks were coronally and semi-serially sectioned in 6 µm to analyze hematoxylin and eosin (HE), immunohistochemistry and *Picrosirius red.*

A photomicrograph of histological sections with HE was taken with an optical microscope coupled to a camera. The images were obtained with a 40× objective, recorded in a TIF file and analyzed in the IMAGE J program (Image Analyzer Program, Ottawa, Ontario Canada).

A histopathological analysis was performed following the inflammation quality guidelines and tissue cellularity pattern from the margin of the bone defect to the inflammatory infiltrate. The mean of inflammatory cells (IC) was evaluated in hematoxylin-eosin-stained at 40× magnification as follows: low (0 to few inflammatory cells), mild (<25 cells), moderate (25-125 cells) and severe (>125 cells). ^31^ The analysis of the inflammatory process consisted of describing the inflammatory phenomena microscopically observed in tissue sections of each group and postoperative times, and performed according to criteria described in ISO 7405. ^32^

Coronal histological sections in the calvaria were stained with *Picrosirius red* to identify and analyze the quantity and quality of collagen by the birefringence of the organization of its fiber bundles. Four central fields of the defects were analyzed under a polarized light microscope at 200× magnification. The intensity of birefringence from greenish collagen fibers (thin fibers) to yellow and red (thick fibers) was measured using AxioVision software to define the corresponding area (pixel ^2^ ) of these fibers and the total birefringent fibers.

### Immunohistochemical analysis to detect type I collagen

The immunoexpression of type I collagen in tissue in contact with the materials and the control group was verified to assess tissue repair.

After deparaffinization and hydration, the sections were immersed in 0.001 M sodium citrate buffer (pH 6.0) and subjected to microwave treatment for 20 minutes at 90-94ºC. After cooling and inactivating the endogenous peroxidase with 3% hydrogen peroxide, the sections were incubated with the primary anti-collagen-1 antibody (anti-COL-1, Santa Cruz Biotechnology, Santa Cruz, California, USA) at a titration of 1:100, at ٤ºC, for 16 hours. After washing in Tris-HCl buffer, the sections were incubated with biotinylated secondary antibody anti-biotinylated mouse/rat/goat IgG (Kit Dako LSAB+ System-HRP, Agilent’s Dako, Carpinteria, California, USA) for 30 minutes at room temperature. The sections were washed with Tris-HCl buffer and incubated with the streptavidin-peroxidase complex for 30 minutes.

The sections were washed with Tris-HCl buffer, the peroxidase activity was revealed with 3.3-diaminobenzidine solution (Betazoid DAB Chromogen Kit – Biocare Medical, Pacheco, California, USA) for 2-3 minutes and, subsequently, counterstained with hematoxylin. As a negative control, the sections were subjected to the same steps, except for incubation with the primary antibody; at this stage, the sections were incubated with non-immune serum.

Images of the defects were captured with the aid of a camera (DP-71, Olympus – Tokyo, Japan) coupled to a light microscope (Olympus, model BX-51), at a final magnification of 695×. Later, with an image analysis program (Image-Pro Express 6.0, Olympus – Tokyo, Japan), the immunopositive tissues were verified.

### Statistical analysis

All data are expressed as mean and standard deviation (SD) and statistically analyzed by one-way analysis of variance (ANOVA) and Tuckey test. All statistical analyses were performed using SigmaPlot 12.5 (Systat software, CA, USA). The p-value was considered significant at 5%.

## Results

No animal died during the experiments and we did not notice side effects due to endodontics sealers. Two different blind investigators were involved in the data analyses for each specimen.

### Histological analysis - HE staining

**Figure 2:**
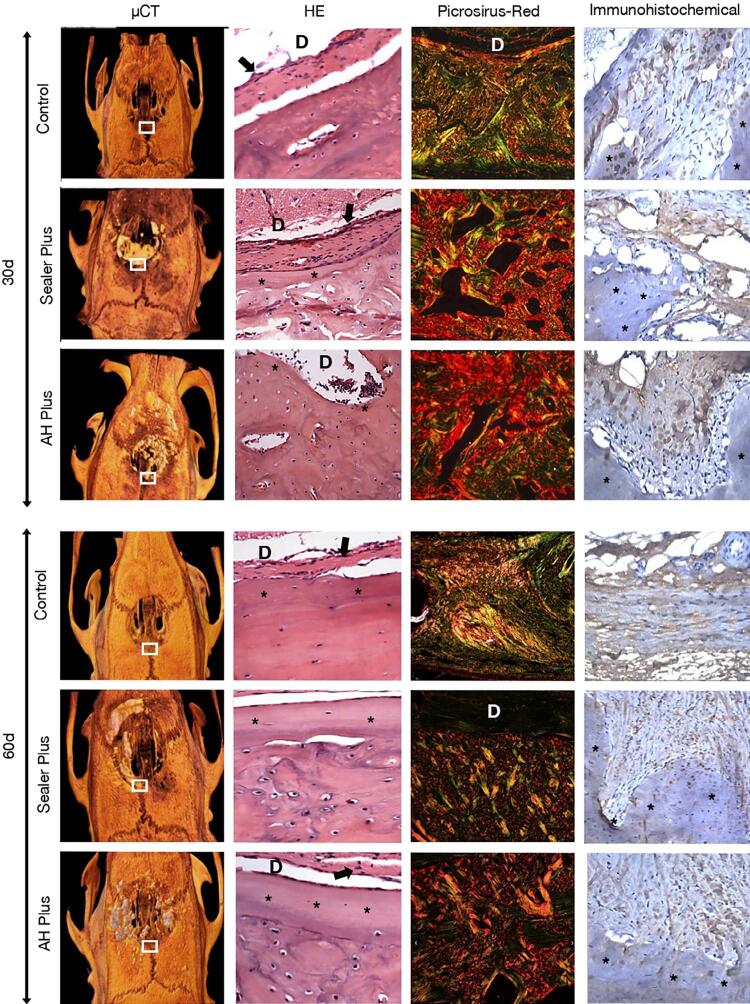
Three-dimensional microtomographic images (µCT; 350×) and photomicrographs of sections (HE, Picrosirius red and Immunohistochemical; 695×) of the portions adjacent to the bone defects (D) performed after 30 days and 60 days. Microtomographic images show all area of bone defect created in the rat calvaria (D). The delineated area (white line) shows the region of the HE, Picrosirius red and Immunohistochemical sections in higher magnification. HE photomicrographs sections showed bone neoformation next to the defects of control group and sealers (asterisks); besides a fibrous capsule was juxtaposed to this bone (arrows); many fibroblasts and few inflammatory cells, especially plasmocytes and lymphocytes, were present in this tissue. Picrosirius red section shows a bundle of collagen fibers (red, orange, green) formed in the adjacent bone. Areas immunomarked (immunohistochemical sections) in brown showed the detection nex to the newly formed bone type I collagen that can be seen in the tissue in contact with the newly formed bone (asterisks)

After 30 days, we observed an intense inflammatory infiltrate in the connective tissue in contact with the created defect. There was no statistically significant difference among the three groups (p>0.05). At 60 days, the control group reduced inflammatory infiltrate, while the Sealer Plus and AH Plus groups maintained an intense inflammatory infiltrate (p<0.05) ( Figure 2 ; Table 1 ).

**Table 1:** Means of inflammatory cells and immunopositive areas in the groups with application of endodontic sealers

Period	Material	Inflammatory cells	Immunopositive areas
		Mean ±SD	Mean ±SD
30 days	Control	142 (±41) ^a^	40 (±13) ^a^
	Sealer Plus	168 (±39) ^a^	39 (±18) ^a^
	AH Plus	154 (±39) ^a^	36 (±17) ^a^
			
60 days	Control	68 (±34) ^a^	23 (±7) ^a^
	Sealer Plus	239 (±85) ^b^	23 (±9) ^a^
	AH Plus	169 (±64) ^b^	25 (±9) ^a^

Standard deviations in parentheses

Different letters indicate differences between groups for the same period (ANOVA and Tuckey tests p<0.05)

Bone neoformation was close to the material at 30 and 60 days, mainly in the Sealer Plus group. In the control group, we observed no bone tissue formation while adjacent to the AH Plus, and bone neoformation was lower than Sealer Plus. In the groups with sealers, we observed a greater formation of bone tissue at 60 days compared to the period of 30 days. During this period, Sealer Plus induced more bone neoformation compared to AH Plus.

### Histological analysis - *Picrosirius* red staining

**Figure 3:**
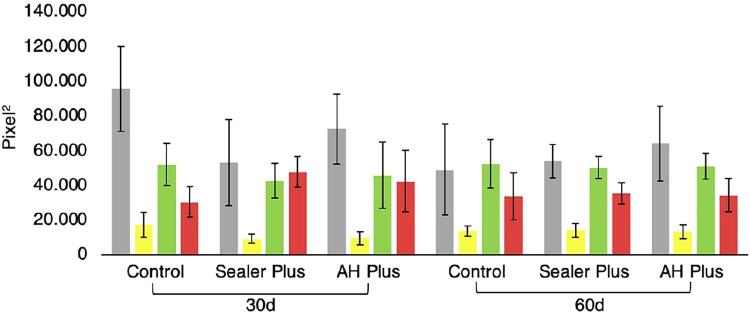
Quantification of collagen fiber bundles by the Picrosirius-polarization method after 30 and 60 days formed in the area next to the defects. Means and standard deviation of birefringence intensity (pixels) measured from the total area of collagen fibers (represented in the gray bars) and yellow, green and red collagen fibers (represented in the bars with the respective colors). Symbols (asterisk or hashtag) indicate a statistically significant difference (p<0.05)

All groups presented birefringence for greenish, yellow and red fibers, showing the matrix maturation dynamics ( Figure 2 and 3 ). Considering the total fibers, the control group showed an increase in the amount of matrix 30 days after surgery compared to 60 days after surgery. No statistically significant difference was detected between groups considering green and yellow fibers in all periods (p>0.05). Considering the red fibers, the control group showed a significant decrease compared to the Sealer Plus group on day 30 (p<0.05).

### Immunohistochemical analysis

After 30 and 60 days, we observed immunopositive areas for type I collagen more frequently in the groups with AH Plus or Sealer Plus than in the control group ( Figure 2 and 3 ). However, this parameter did not show a statistically significant difference between the three groups in both periods (p<0.05) ( Figure 2 ).

### Microtomographic Analysis

**Figure 4:**
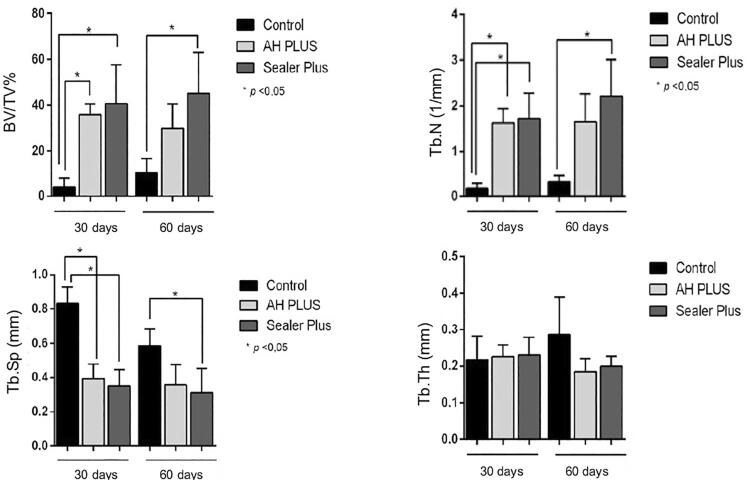
Histomorphometric analysis of the newly formed bone volume (BV/TV%), and the number (Tb.N), separation (Tb.Sp) and thickness of bone trabeculae (Tb.Th) in both experimental periods (30 and 60 days)

At 30 days, the newly formed bone volume and number of bone trabeculae in the Sealer Plus and AH Plus groups were higher compared to the control group (p<0.05). At 60 days, Sealer Plus had more bone neoformation compared to the control group (p<0.05), but it did not differ from AH Plus (p>0.05). Consequently, both sealers analyzed at 30 days showed a gradual decrease in trabecular separation, with statistical difference compared to the control group (p<0.05). This same parameter showed a statistical difference in the Sealer Plus group at 60 days compared to the control group (p<0.05). Regarding trabecular thickness, there was no statistical difference between the groups in both periods analyzed (p>0.05). ( Figures 2 and 4 ).

## Discussion

An ideal endodontic sealer must have adequate physicochemical properties, antimicrobial activity and biocompatibility, since they will be in contact with the periapical tissues. ^13 , 33 , 34^ Sealer extrusion may happen in some clinical situations, such as in apical root resorption. ^7^ In these cases, the material must not interfere or aid in the repair process, including bone remodeling. Overfilled material may act as a foreign body and the tissue response depends on its composition. ^8^ Previous research already studied the biocompatibility, cytotoxicity and bioactive potential of Sealer Plus, ^21 , 25^ however, the influence of this material on bone repair was unknown. Thus, our study evaluated the biological response of Sealer Plus in direct contact with the bone, simulating an apical extrusion. Based on the results, the Sealer Plus showed a similar result to the AH Plus regarding bone repair capacity within the evaluated periods. Therefore, there was bone repair in the presence of resin-based endodontic sealers, thus rejecting the null hypothesis.

Our data showed that, despite Sealer Plus and AH Plus induced a more intense inflammatory after 60 days, these materials allow the healing process. Large areas of bone neoformation occurred in contact with the materials in both experimental periods. Since the amount of bone repair was lower in the control group, we can state that sealers can modulate the formation of this tissue. Furthermore, we verified a greater bone formation adjacent to Sealer Plus at 60 days compared to AH Plus. These findings are reinforced by the increased amount of collagen fiber and type I collagen present in the tissue next to the materials.

Studies show that biological properties are associated with the composition of the materials. The composition of Sealer Plus is similar to AH Plus, with the addition of calcium hydroxide. The literature reports that calcium hydroxide has essential effects on tissue, promoting an alkaline pH, antimicrobial effect and accelerating tissue repair. ^35 , 36^ Besides, the presence of calcium hydroxide induces mineralization by dissociation of calcium (Ca+) and hydroxyl ions (OH-), activating alkaline phosphatase and inducing cell differentiation. The release of calcium ions also allows the activation of calcium-dependent ATPase and the formation of calcite microcrystals that will initiate the tissue mineralization process. ^37^ Furthermore, the alkaline pH may stimulate the recruitment of inflammatory cells and the production of cytokines that enhance leukocyte adhesion to endothelial walls, potentiation of neutrophils, and differentiation of plasma cells. The inflammatory process involves a complex and coordinated cascade of cellular and molecular events that culminate with superficial necrosis, a scaffold for healing process and mineralization. ^38^

The presence of calcium hydroxide in the composition of Sealer Plus may accelerate the repair process, increasing the volume of bone formation. A previous study showed that adding calcium hydroxide to AH Plus improved the biological properties of this sealer ^35^ without affecting part of its physical properties. ^34^ These findings corroborate the ability of calcium hydroxide to improve the biological properties of endodontic materials.

Analysis of the quantity of birefringent collagen showed thicker bundles of fibers in all groups after the experimental periods, predominantly in red. This coloring was related to the presence of mature collagen, ^39^ characterizing the repair of the tissue. We observed a significant amount of red collagen adjacent to the Sealer Plus and AH Plus after 30 days compared to the control group (p<0.05). Despite many inflammatory cells, the repair process in the adjacent tissue may have been initiated faster than the control group. This hypothesis is reinforced for the immunohistochemical analysis, since immunopositive areas for type I collagen were more frequent in the Sealer Plus and AH Plus groups. A previous study showed that cell proliferation and adhesion of extracellular matrix proteins, such as type I collagen, in the migration process are involved in the colonization of cells to tissues. Thus, the expression of these proteins is crucial for the repair process. ^40^ The repair process in the adjacent tissue occurs because both materials caused a reduced inflammatory reaction, culminating with bone neoformation as seen after 30 and 60 days.

During root canal filling, the materials used may come into contact with the periapical tissue. Among the properties of the materials, biocompatibility is an essential characteristic. ^41 , 42^ Endodontic sealers should allow or promote the resolution of periapical inflammatory and/or infections. ^41^ The healing process is dynamic and involves different stages. Initially, the inflammatory process decreases, followed by the remodeling of the tissue and the formation of collagen. Finally, when bone loss occurs, there will be the neoformation of this tissue. According to our results, both Sealer Plus and AH Plus modulate the repair process, since they allow the formation of collagen and bone faster than the control group.

Among the limitations of our study, we emphasize that the results must be considered before being extrapolated to humans, since *Rattus norvegicus* is a different species. However, this study helps to further assess Sealer Plus participation in the bone repair process.

## Conclusion

The results showed that Sealer Plus was similar to AH Plus in the inflammatory process, in the maturation of collagen fibers and in bone neoformation in both evaluated periods. Therefore, Sealer Plus and AH Plus can be considered biocompatible materials, modulating the bone repair process. Clinically, both materials are indicated in cases with risk of overfilling.

## References

[1] Sousa CJ , Loyola AM , Versiani MA , Biffi JC , Oliveira RP , Pascon EA . A comparative histological evaluation of the biocompatibility of materials used in apical surgery . Int Endod J . 2004 ; 37 ( 11 ): 738 - 48 . doi: 10.1111/j.1365-2591.2004.00861.x 10.1111/j.1365-2591.2004.00861.x 15479256

[2] Schilder H . Filling root canals in three dimensions . Dent Clin North Am . 1967 : 723 - 44 . 5262492

[3] Sundqvist G , Figdor D , Persson S , Sjögren U . Microbiologic analysis of teeth with failed endodontic treatment and the outcome of conservative re-treatment . Oral Surg Oral Med Oral Pathol Oral Radiol Endod . 1998 ; 85 ( 1 ): 86 - 93 . doi: 10.1016/s1079-2104(98)90404-8 10.1016/s1079-2104(98)90404-8 9474621

[4] Flores DS , Rached FJ Jr , Versiani MA , Guedes DF , Sousa-Neto MD , Pécora JD . Evaluation of physicochemical properties of four root canal sealers . Int Endod J . 2011 ; 44 ( 2 ): 126 - 35 . doi: 10.1111/j.1365-2591.2010.01815.x 10.1111/j.1365-2591.2010.01815.x 21091494

[5] Ricucci D , Langeland K . Apical limit of root canal instrumentation and obturation, part 2. A histological study . Int Endod J . 1998 ; 31 ( 6 ): 394 - 409 . doi: 10.1046/j.1365-2591.1998.00183.x 10.1046/j.1365-2591.1998.00183.x 15551607

[6] Schaeffer MA , White RR , Walton RE . Determining the optimal obturation length: a meta-analysis of literature . J Endod . 2005 ; 31 ( 4 ): 271 - 4 . doi: 10.1097/01.don.0000140585.52178.78 10.1097/01.don.0000140585.52178.78 15793382

[7] Ricucci D , Rôças IN , Alves FR , Loghin S , Siqueira JF Jr . Apically extruded sealers: fate and influence on treatment outcome . J Endod . 2016 ; 42 ( 2 ): 243 - 9 . doi: 10.1016/j.joen.2015.11.020 10.1016/j.joen.2015.11.020 26725179

[8] Pascon EA , Leonardo MR , Safavi K , Langeland K . Tissue reaction to endodontic materials: methods, criteria, assessment, and observations . Oral Surg Oral Med Oral Pathol . 1991 ; 72 ( 2 ): 222 - 37 . doi: 10.1016/0030-4220(91)90168-c 10.1016/0030-4220(91)90168-c 1833711

[9] Duarte MA , Ordinola-Zapata R , Bernardes RA , Bramante CM , Bernardineli N , Garcia RB , et al . Influence of calcium hydroxide association on the physical properties of AH Plus . J Endod . 2010 ; 36 ( 6 ): 1048 - 51 . doi: 10.1016/j.joen.2010.02.007 10.1016/j.joen.2010.02.007 20478463

[10] Marciano MA , Guimarães BM , Ordinola-Zapata R , Bramante CM , Cavenago BC , Garcia RB , et al . Physical properties and interfacial adaptation of three epoxy resin-based sealers . J Endod . 2011 ; 37 ( 10 ): 1417 - 21 . doi: 10.1016/j.joen.2011.06.023 10.1016/j.joen.2011.06.023 21924194

[11] Amoroso-Silva PA , Guimarães BM , Marciano MA , Duarte MA , Cavenago BC , Ordinola-Zapata , et al . Microscopic analysis of the quality of obturation and physical properties of MTA Fillapex . Microsc Res Tech . 2014 ; 77 ( 12 ): 1031 - 6 . doi: 10.1002/jemt.22432 10.1002/jemt.22432 25209870

[12] Gomes-Filho JE , Gomes BP , Zaia AA , Ferraz CR , Souza-Filho FJ . Evaluation of the biocompatibility of root canal sealers using subcutaneous implants . J Appl Oral Sci . 2007 ; 15 ( 3 ): 186 - 94 . doi: 10.1590/s1678-77572007000300007 10.1590/S1678-77572007000300007 PMC4327465 19089128

[13] Scarparo RK , Grecca FS , Fachin EV . Analysis of tissue reactions to methacrylate resin-based, epoxy resin-based, and zinc oxide-eugenol endodontic sealers . J Endod . 2009 ; 35 ( 2 ): 229 - 32 . doi: 10.1016/j.joen.2008.10.025 10.1016/j.joen.2008.10.025 19166779

[14] Santos J , Tjäderhane L , Ferraz C , Zaia A , Alves M , De Goes M , et al . Long-term sealing ability of resin-based root canal fillings . Int Endod J . 2010 ; 43 ( 6 ): 455 - 60 . doi: 10.1111/j.1365-2591.2010.01687.x 10.1111/j.1365-2591.2010.01687.x 20536572

[15] Vasconcelos BC , Bernardes RA , Duarte MA , Bramante CM , Moraes IG . Apical sealing of root canal fillings performed with five different endodontic sealers: analysis by fluid filtration . J Appl Oral Sci . 2011 ; 19 ( 4 ): 324 - 8 . doi: 10.1590/s1678-77572011005000005 10.1590/S1678-77572011005000005 PMC4223782 21655776

[16] Rezende GC , Massunari L , Queiroz IO , Gomes JE Filho , Jacinto RC , Lodi CS , et al . Antimicrobial action of calcium hydroxide-based endodontic sealers after setting, against *E. faecalis* biofilm . Braz Oral Res . 2016 ; 30 : S1806-83242016000100228 . doi: 10.1590/1807-3107BOR-2016.vol30.0038 10.1590/1807-3107BOR-2016.vol30.0038 26981759

[17] Leonardo MR , Silva LA , Almeida WA , Utrilla LS . Tissue response to an epoxy resin-based root canal sealer . Endod Dent Traumatol . 1999 ; 15 ( 1 ): 28 - 32 . doi: 10.1111/j.1600-9657.1999.tb00745.x 10.1111/j.1600-9657.1999.tb00745.x 10219151

[18] Leonardo MR , Flores DS , Paula e Silva FW , Toledo Leonardo R , Silva LA . A comparison study of periapical repair in dogs’ teeth using RoekoSeal and AH plus root canal sealers: a histopathological evaluation . J Endod . 2008 ; 34 ( 7 ): 822 - 5 . doi: 10.1016/j.joen.2008.03.029 10.1016/j.joen.2008.03.029 18570987

[19] Tanomaru-Filho M , Tanomaru JM , Leonardo MR , Silva LA . Periapical repair after root canal filling with different root canal sealers . Braz Dent J . 2009 ; 20 ( 5 ): 389 - 95 . doi: 10.1590/s0103-64402009000500006 10.1590/s0103-64402009000500006 20126907

[20] Assmann E , Böttcher DE , Hoppe CB , Grecca FS , Kopper PM . Evaluation of bone tissue response to a sealer containing mineral trioxide aggregate . J Endod . 2015 ; 41 ( 1 ): 62 - 6 . doi: 10.1016/j.joen.2014.09.019 10.1016/j.joen.2014.09.019 25447498

[21] Cintra LT , Benetti F , Azevedo Queiroz ÍO , Ferreira LL , Massunari L , Bueno CR , et al . Evaluation of the cytotoxicity and biocompatibility of new resin epoxy-based endodontic saler containing calcium hydroxide . J Endod . 2017 ; 43 ( 12 ): 2088 - 2092 . doi: 10.1016/j.joen.2017.07.016 10.1016/j.joen.2017.07.016 29032822

[22] Vertuan GC , Duarte MA , Moraes IG , Piazza B , Vasconcelos BC , Alcalde MP , et al . Evaluation of physicochemical properties of a new root canal sealer . J Endod . 2018 ; 44 ( 3 ): 501 - 5 . doi: 10.1016/j.joen.2017.09.017 10.1016/j.joen.2017.09.017 29254816

[23] Piai GG , Duarte MA , Nascimento AL , Rosa RA , Só MV , Vivan RR . Penetrability of a new endodontic sealer: a confocal laser scanning microscopy evaluation . Microsc Res Tech . 2018 ; 81 ( 11 ): 1246 - 9 . doi: 10.1002/jemt.23129 10.1002/jemt.23129 30295382

[24] Furtado TC , Bem IA , Machado LS , Pereira JR , Só MV , Rosa RA . Intratubular penetration of endodontic sealers depends on the fluorophore used for CLSM assessment . Microsc Res Tech . 2021 ; 84 ( 2 ): 305 - 12 . doi: 10.1002/jemt.23589 10.1002/jemt.23589 32914923

[25] Tanomaru-Filho M , Cristine Prado M , Torres FF , Viapiana R , Pivoto-João MM , Guerreiro-Tanomaru JM . Physicochemical properties and bioactive potential of a new epoxy resin-based root canal sealer . Braz Dent J . 2019 ; 30 ( 6 ): 563 - 8 . doi: 10.1590/0103-6440201802861 10.1590/0103-6440201802861 31800750

[26] Silva EJ , Hecksher F , Vieira VT , Vivan RR , Duarte MA , Brasil SC , et al . Cytotoxicity, antibacterial and physicochemical properties of a new epoxy resin-based endodontic sealer containing calcium hydroxide . J Clin Exp Dent . 2020 ; 12 ( 6 ): e533 - 9 . doi: 10.4317/jced.56534 10.4317/jced.56534 PMC7335608 32665811

[27] Benetti F , Azevedo Queiroz IO , Oliveira PH , Conti LC , Azuma MM , Oliveira SH , et al . Cytotoxicity and biocompatibility of a new bioceramic endodontic sealer containing calcium hydroxide . Braz Oral Res . 2019 ; 33 : e042 . doi: 10.1590/1807-3107bor-2019.vol33.0042 10.1590/1807-3107bor-2019.vol33.0042 31508725

[28] Kilkenny C , Browne W , Cuthill IC , Emerson M , Altman DG ; NC3Rs Reporting Guidelines Working Group . Animal research: reporting *in vivo* experiments: the ARRIVE guidelines . Br J Pharmacol . 2010 ; 160 ( 7 ): 1577 - 9 . doi: 10.1111/j.1476-5381.2010.00872.x 10.1111/j.1476-5381.2010.00872.x PMC2936830 20649561

[29] Nagendrababu V , Kishen A , Murray PE , Nekoofar MH , Figueiredo JA , Priya E , at al . PRIASE 2021 guidelines for reporting animal studies in Endodontology: a consensus-based development . Int Endod J . 2021 ; 54 ( 6 ): 848 - 57 . doi: 10.1111/iej.13477 10.1111/iej.13477 33450080

[30] Luvizuto ER , Oliveira JC , Gomes-Ferreira PH , Pereira CC , Faverani LP , Antoniali C , et al . Immunohistochemical response in rats of beta-tricalcium phosphate (TCP) with or without BMP-2 in the production of collagen matrix critical defects . Acta Histochem . 2017 ; 119 ( 3 ): 302 - 8 . doi: 10.1016/j.acthis.2017.02.006 10.1016/j.acthis.2017.02.006 28262327

[31] Cosme-Silva L , Santos AF , Lopes CS , Dal-Fabbro R , Benetti F , Gomes-Filho JE , et al . Cytotoxicity, inflammation, biomineralization, and immunoexpression of IL-1β and TNF-α promoted by a new bioceramic cement . J Appl Oral Sci . 2020 ; 28 : e20200033 . doi: 10.1590/1678-7757-2020-0033 10.1590/1678-7757-2020-0033 PMC7406194 32785523

[32] International Standards Organization . ISO 7405. Dentistry - preclinical evaluation of biocompatibility of medical devices used in dentistry - test methods for dental materials; 1997 . Geneve : ISO ; 2018 .

[33] Cintra LT , Bernabé PF , Moraes IG , Gomes-Filho JE , Okamoto T , Consolaro A , et al . Evaluation of subcutaneous and alveolar implantation surgical sites in the study of the biological properties of root-end filling endodontic materials . J Appl Oral Sci . 2010 ; 18 ( 1 ): 75 - 82 . doi: 10.1590/s1678-77572010000100013 10.1590/S1678-77572010000100013 PMC5349029 20379685

[34] Marín-Bauza GA , Silva-Sousa YT , Cunha SA , Rached-Junior FJ , Bonetti-Filho I , Sousa-Neto MD , et al . Physicochemical properties of endodontic sealers of different bases . J Appl Oral Sci . 2012 ; 20 ( 4 ): 455 - 61 . doi: 10.1590/s1678-77572012000400011 10.1590/S1678-77572012000400011 PMC3881818 23032208

[35] Duarte MA , O Demarchi AC , Moraes IG . Determination of pH and calcium ion release provided by pure and calcium hydroxide-containing AHPlus . Int Endod J . 2004 ; 37 ( 1 ): 42 - 5 . doi: 10.1111/j.1365-2591.2004.00756.x 10.1111/j.1365-2591.2004.00756.x 14718056

[36] Oliveira RL , Oliveira RS Filho , Gomes HC , Franco MF , Enokihara MM , Duarte MA . Influence of calcium hydroxide addition to AH Plus sealer on its biocompatibility . Oral Surg Oral Med Oral Pathol Oral Radiol Endod . 2010 ; 109 ( 1 ): e50 - 4 . doi: 10.1016/j.tripleo.2009.08.026 10.1016/j.tripleo.2009.08.026 20123371

[37] Seux D , Couble ML , Hartmann DJ , Gauthier JP , Magloire H . Odontoblast-like cytodifferentiation of human dental pulp cells *in vitro* in the presence of a calcium hydroxide-containing cement . Arch Oral Biol . 1991 ; 36 ( 2 ): 117 - 28 . doi: 10.1016/0003-9969(91)90074-5 10.1016/0003-9969(91)90074-5 2059161

[38] Estrela C , Sydney GB , Bammann LL , Felippe O Júnior . Mechanism of action of calcium and hydroxyl ions of calcium hydroxide on tissue and bacteria . Braz Dent J . 1995 ; 6 ( 2 ): 85 - 90 . 8688662

[39] Junqueira LC , Montes GS , Krisztán RM . The collagen of the vertebrate peripheral nervous system . Cell Tissue Res . 1979 ; 202 ( 3 ): 453 - 60 . doi: 10.1007/BF00220437 10.1007/BF00220437 519713

[40] Rodríguez-Lozano FJ , García-Bernal D , Oñate-Sánchez RE , Ortolani-Seltenerich PS , Forner L , Moraleda JM . Evaluation of cytocompatibility of calcium silicate-based endodontic sealers and their effects on the biological responses of mesenchymal dental stem cells . Int Endod J . 2017 ; 50 ( 1 ): 67 - 76 . doi: 10.1111/iej.12596 10.1111/iej.12596 26660310

[41] Johnson W , Kulild JC , Tay F . Obturation of the cleaned and shaped root canal system . In: Hargreaves KM , Berman LH , editors . Cohen’s pathways of the pulp . 11rd ed. St. Louis, USA : Elsevier , 2017 . p. 280 - 322 .

[42] Lee BN , Hong JU , Kim SM , Jang JH , Chang HS , Hwang YC , et al . Anti-inflammatory and osteogenic effects of calcium silicate-based root canal sealers . J Endod . 2019 ; 45 ( 1 ): 73 - 8 . doi: 10.1016/j.joen.2018.09.006 10.1016/j.joen.2018.09.006 30558800

